# A Rare Cause of Granulomatous Lymphadenitis: Tularemia

**DOI:** 10.1590/0037-8682-0066-2025

**Published:** 2025-09-29

**Authors:** Celal Yazıcı

**Affiliations:** 1 Osmaniye State Hospital, Department of Radiology, Osmaniye, Turkey.

A 49-year-old woman presented with fever, persistent swelling, and redness on the left side of her neck that had persisted for 1 month. Laboratory results showed an elevated leukocyte count (13.5×10³/µL) and a high C-reactive protein level (103 mg/L).

**FIGURE 1:**
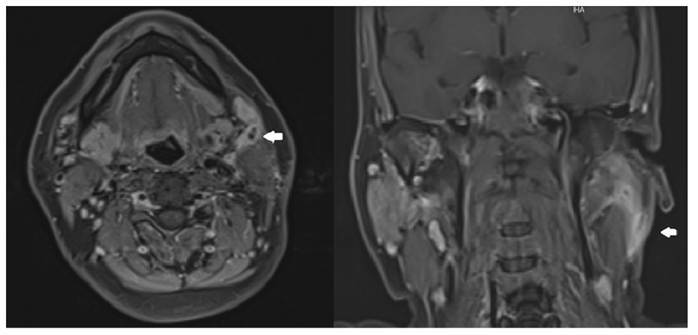
Contrast-enhanced neck computed tomography showing multiple necrotic lymph nodes and thickened, hyper-enhanced skin (white arrows).

Contrast-enhanced neck computed tomography revealed necrotic lymph nodes, increased skin thickness, and contrast enhancement at cervical levels 2 and 3 (Figure 1).

**FIGURE 2:**
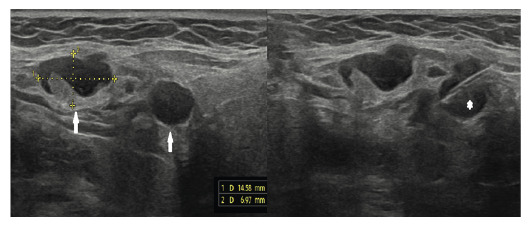
Ultrasound image demonstrating thickened cortex and reactive lymph nodes (white arrows). Fine-needle aspiration biopsy was performed (white star).

Ultrasound of the affected area revealed reactive and necrotic lymph nodes with thickened cortices. The surrounding tissues exhibited increased echogenicity, indicating inflammation. Fine-needle aspiration biopsy was performed (Figure 2).

Histopathological examination confirmed granulomatous lymphadenitis. A tube agglutination test for *Francisella tularensis* yielded positive results, supporting the diagnosis of tularemia. The patient was treated with appropriate antibiotics, which resulted in significant clinical and laboratory improvement.

Tularemia is an infectious disease caused by the Gram-negative bacterium *Francisella tularensis*. This report presents a rare case of granulomatous lymphadenitis secondary to tularemia.

Tularemia-associated lymphadenopathy can manifest as abscess granuloma formation. Lymph node involvement typically occurs approximately 1 week after the appearance of skin lesions^1^. 

Imaging studies are crucial for the diagnosis. The main radiological findings included cervical lymphadenopathy and skin edema. Lymph nodes may show necrosis or suppuration, as observed in this case^2^.

Diagnostic criteria include a single test cutoff value (e.g., ≥1:160) or a fourfold titer increase between acute and convalescent samples^3^. 

Tularemia should be considered in the differential diagnosis of necrotic lymphadenopathy. Imaging, combined with serological and microbiological testing, plays a critical role in the timely diagnosis of this disease^4^.
